# Closing delivery gaps in the treatment of tuberculosis infection: Lessons from implementation research in Peru

**DOI:** 10.1371/journal.pone.0247411

**Published:** 2021-02-19

**Authors:** Courtney M. Yuen, Ana Karina Millones, Daniela Puma, Judith Jimenez, Jerome T. Galea, Roger Calderon, Gabriela S. Pages, Meredith B. Brooks, Leonid Lecca, Tom Nicholson, Mercedes C. Becerra, Salmaan Keshavjee

**Affiliations:** 1 Division of Global Health Equity, Brigham and Women’s Hospital, Boston, MA, United States of America; 2 Department of Global Health and Social Medicine, Harvard Medical School, Boston, MA, United States of America; 3 Harvard Medical School Center for Global Health Delivery, Harvard Medical School, Boston, MA, United States of America; 4 Socios En Salud Sucursal Perú, Lima, Peru; 5 School of Social Work, University of South Florida, Tampa, FL, United States of America; 6 College of Public Health, University of South Florida, Tampa, FL, United States of America; 7 Duke Center for International Development, Sanford School of Public Policy, Duke University, Durham, NC, United States of America; 8 Advance Access & Delivery, Durham, NC, United States of America; Agencia de Salut Publica de Barcelona, SPAIN

## Abstract

**Background:**

Targeted testing and treatment of TB infection to prevent disease is a pillar of TB elimination. Despite recent global commitments to greatly expand access to preventive treatment for TB infection, there remains a lack of research on how best to expand preventive treatment programs in settings with high TB burdens.

**Methods:**

We conducted implementation research in Lima, Peru, around a multifaceted intervention to deliver TB preventive treatment to close contacts of all ages, health care workers, and people in congregate settings. Key interventions included use of the interferon gamma release assay (IGRA), specialist support for generalist physicians at primary-level health facilities, and treatment support by community health workers. We applied a convergent mixed methods approach to evaluate feasibility and acceptability based on a care cascade framework.

**Findings:**

During April 2019-January 2020, we enrolled 1,002 household contacts, 148 non-household contacts, 107 residents and staff of congregate settings, and 357 health care workers. Cumulative completion of the TB preventive care cascade was 34% for contacts <5 years old, 28% for contacts 5–19 years old, 18% for contacts ≥20 years old, 0% for people in congregate settings, and 4% of health care workers. IGRA testing was acceptable to adults exposed to TB. Preventive treatment was acceptable to contacts, but less acceptable to physicians, who frequently had doubts about prescribing preventive treatment for adults. Community-based treatment support was both acceptable and feasible, and periodic home-visits or calls were identified as facilitators of adherence.

**Conclusions:**

We attempted to close the gap in TB preventive treatment in Peru by expanding preventive services to adult contacts and other risk groups. While suboptimal, care cascade completion for adult contacts was consistent with what has been observed in high-income settings. The major losses in the care cascade occurred in completing evaluations and having doctors prescribe preventive treatment.

## Introduction

Globally, more than 10 million people develop tuberculosis (TB) each year and 1.5 million die—more than 4,000 people each day. Despite the availability of curative treatment for TB disease since the late 1940s, and preventive treatment for TB infection since the late 1950s, rates of TB have dropped at an extremely slow 1.5 to 1.8 percent per annum over the last 20 years [1]. The Zero TB Initiative is an emerging network of coalitions seeking to rapidly drive down TB case rates in geographically defined zones by deploying the evidence-based strategy of simultaneously increasing case-finding, access to treatment for all forms of TB disease, and access to TB preventive treatment to prevent development of TB disease [1]. Widespread access to TB preventive treatment until recently has not been the norm outside of high-income countries, but it is necessary to achieve significant reductions in the global TB burden [2,3].

Preventive treatment of TB infection in close contacts and high-risk communities is a pillar of TB elimination [4]. In high-income countries, TB preventive treatment has been targeted to multiple groups at risk, including people who have been in close contact with someone with TB, people living with HIV, health care workers, and people in prisons, among others [5]. In contrast, following decades of “minimalist” guidance from international health agencies to low- and middle-income countries [6], TB programs in countries with high TB burdens have focused TB preventive treatment only on child contacts <5 years old [7] and people living with HIV [8], the groups with perceived highest risk. However, these two groups contribute relatively little to the global TB burden, accounting for around 10% of notified cases in 2019 [9].

In 2018, World Health Organization guidance for all countries, regardless of TB burden or economic status, came into line with best practices that call for all locally relevant populations with elevated risk of TB to be screened and treated for TB infection [10,11]. At the United Nations High Level Meeting on tuberculosis, countries of the world committed to ensure that 30 million people would receive preventive treatment by 2022 [12]. However, only a fraction of that goal had been achieved by 2020 [9]. Implementation research is lacking on effective strategies to achieve changes in local programs to close this TB preventive treatment gap in low- and middle-income settings [13,14].

A coalition of local, national, and international partners launched a TB elimination initiative in Lima, Peru, a middle-income setting with moderate incidence and low HIV prevalence. Key components of the initiative included community-based active case-finding with x-ray vans [15] and reaching known high-risk populations with TB infection testing and preventive treatment. As part of the latter component, we sought to explore strategies for changing practices around testing and treatment for TB infection, attempting to close the preventive treatment gap by expanding its use beyond only young child contacts and people living with HIV. We conducted implementation research around a multifaceted intervention to provide preventive treatment to older contacts, health care workers, and people living in congregate settings.

## Materials and methods

### Study population and setting

We evaluated a multifaceted intervention designed to change practices around the use of TB preventive treatment, which was implemented in the eastern region of Carabayllo District (population 261,000) of Lima, Peru. The evaluation included participants enrolled during April 2019-January 2020.

Peru is a middle-income country with an estimated TB incidence of 119 per 100,000 and low HIV prevalence in the general population [9]. In the 2015 national drug resistance survey, 7.3% of patients with TB and no prior treatment history had multidrug-resistant TB [16]. TB treatment and contact management takes place in primary-level health facilities staffed by generalist doctors. The tuberculin skin test (TST) rather than the blood-based interferon gamma release assay (IGRA) is used to test for TB infection.

National TB guidelines [17] mandate preventive treatment with 6 months of isoniazid following rule-out of active TB disease for five groups: (a) children <5 years old who are contacts of patients with pulmonary TB, regardless of TST result; (b) people 5–19 years who are contacts of pulmonary patients with pulmonary TB and who have TST results of ≥10 mm induration; (c) people living with HIV, regardless of TST result; (d) health care workers with TST conversion; and (e) people with certain medical comorbidities, based on clinician judgement. The guidelines neither mandate nor prohibit preventive treatment for adult close contacts, but few are typically treated [18]. As isoniazid is the only preventive treatment medication used in this setting, the guidelines recommend that preventive treatment not be given to contacts of patients with drug-resistant TB because of presumed resistant infection, although new guidance to use levofloxacin is currently under consideration.

### Intervention approach

In 2019, the non-governmental organization Socios En Salud in collaboration with the North Lima health authority (DIRIS Lima Norte) developed an intervention aimed to promote TB preventive treatment for close contacts of all ages, as well as for health care workers and people in congregate settings that had recently had TB cases. The intervention approach was designed to address barriers encountered in 2015–2016 during a previous effort to improve preventive treatment practices among child contacts in the same population [19] and informed by local qualitative observations [20,21] (S1 Table). The approach was developed with Socios En Salud staff who participated in the 2015–2016 intervention, doctors and administrators at the public health facilities, the investigators, and local residents.

**Fig 1:**
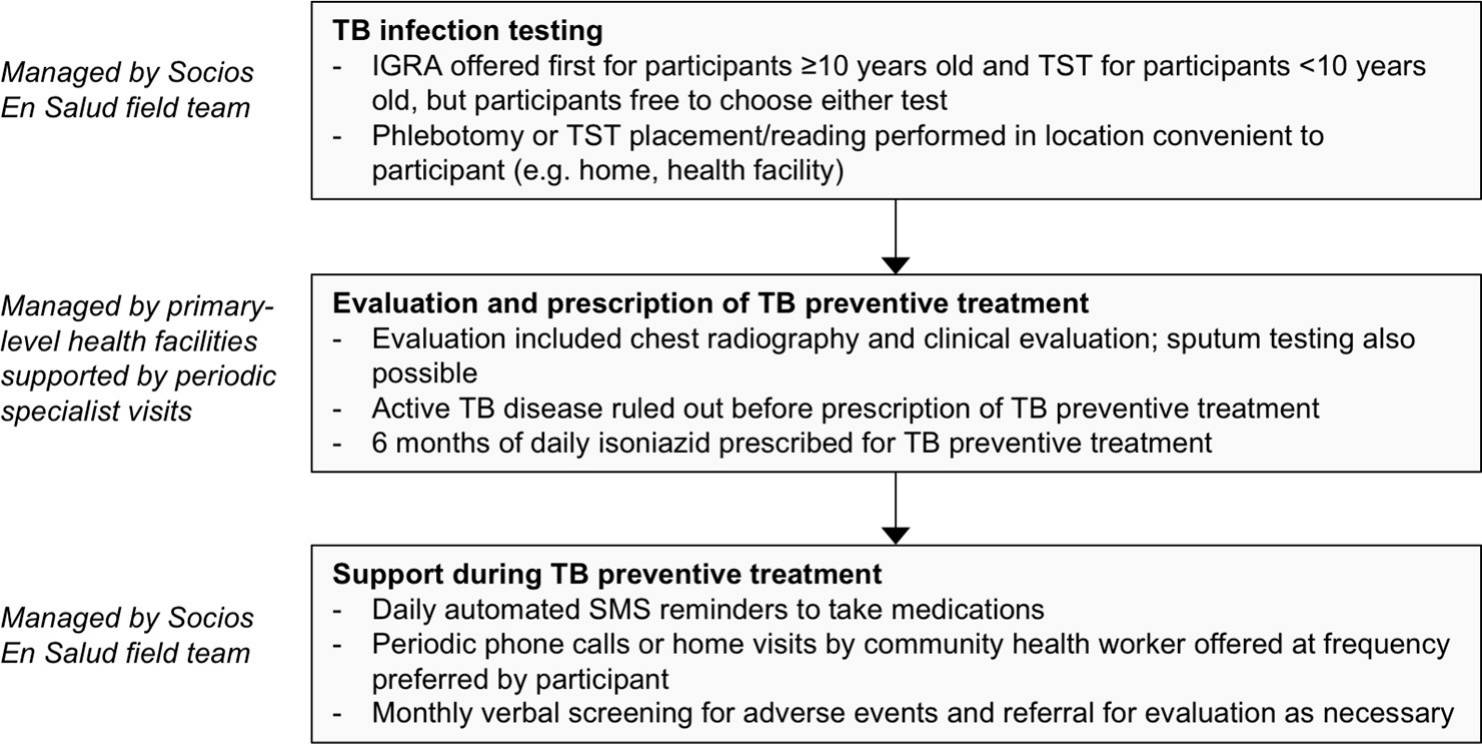
Key procedures for intervention participants. Abbreviations: TB = tuberculosis, IGRA = interferon gamma release assay, TST = tuberculin skin test.

A Socios En Salud field team worked with participants in their communities, while health facility clinicians were responsible for all clinical management (Fig 1). Key elements of the intervention included offering testing to all persons in key populations, using IGRA testing (QuantiFERON TB Gold Plus, Qiagen, Germantown, MD), performing tests at locations convenient to participants, contracting specialists (pulmonologist and pediatrician) to visit primary-level health facilities to support generalist doctors in clinical evaluations and management of preventive treatment, and community-based treatment support tailored to participant preferences.

### Intervention participants

A Socios En Salud field team comprising nurses, nurse technicians, and community health workers (community members with basic training on health issues) recruited three target groups of participants: close contacts of TB patients, residents and staff of congregate settings, and health care workers. To identify close contacts, the field team worked with the TB programs at the nine public health facilities in the eastern region of Carabayllo to conduct contact investigations for all patients newly diagnosed with TB; all contacts in this region were thus recruited for the intervention. The congregate setting participants represented a convenience sample, as health facility staff identified for intervention congregate settings that had recently had TB cases, including a drug rehabilitation center, a residential center for the elderly, and a government child-care center. Finally, the staff of the nine health facilities were invited to participate.

### Intervention implementation

The field team counseled potential participants about TB infection and preventive treatment, informing them that if they enrolled, they could access free testing for TB infection and follow-up during treatment, if indicated. People who were currently being treated for TB disease were ineligible for enrollment. Participants <10 years old were recommended to receive a TST due to the difficulty of phlebotomy, while participants 10 years and older were first offered an IGRA. However, participants or their guardians were free to choose either test. The field team performed TST and IGRA phlebotomy in locations convenient to participants (e.g., at home or at a local health facility). TSTs were read 48–72 hours after placement, with an induration of 10 mm or greater considered positive. IGRAs were processed at the Socios En Salud laboratory.

Physicians at the primary-level health facilities were responsible for the diagnosis of TB disease, the decision to prescribe preventive treatment, and the management of treatment. The intervention contracted two specialists (an adult pulmonologist and a pediatric pulmonologist) from hospitals in Lima to periodically visit the health facilities to provide consultations and mentorship to these physicians; a Socios En Salud pediatrician also assisted with consultations. The field team helped participants make appointments for evaluation procedures, which included chest radiography and clinical evaluation, and could include sputum testing.

If TB preventive treatment was prescribed, the field team offered participants follow-up support. Participants were asked whether they would like to receive weekly, biweekly, or monthly phone calls or visits from community health workers, and they were offered free automated daily SMS reminders to take medications (Memora Health, San Francisco, CA). Participants were counseled about potential adverse events and given the phone number of a field team member to call in the event of concerning symptoms. Field team members provided the type of support requested by participants and contacted participants monthly to administer an adverse event questionnaire (S1 Appendix). The questionnaire asked whether participants had experienced specific symptoms consistent with reaction to isoniazid treatment in the past month, and the field team referred participants for evaluation as necessary.

### Mixed-methods evaluation

**Fig 2:**
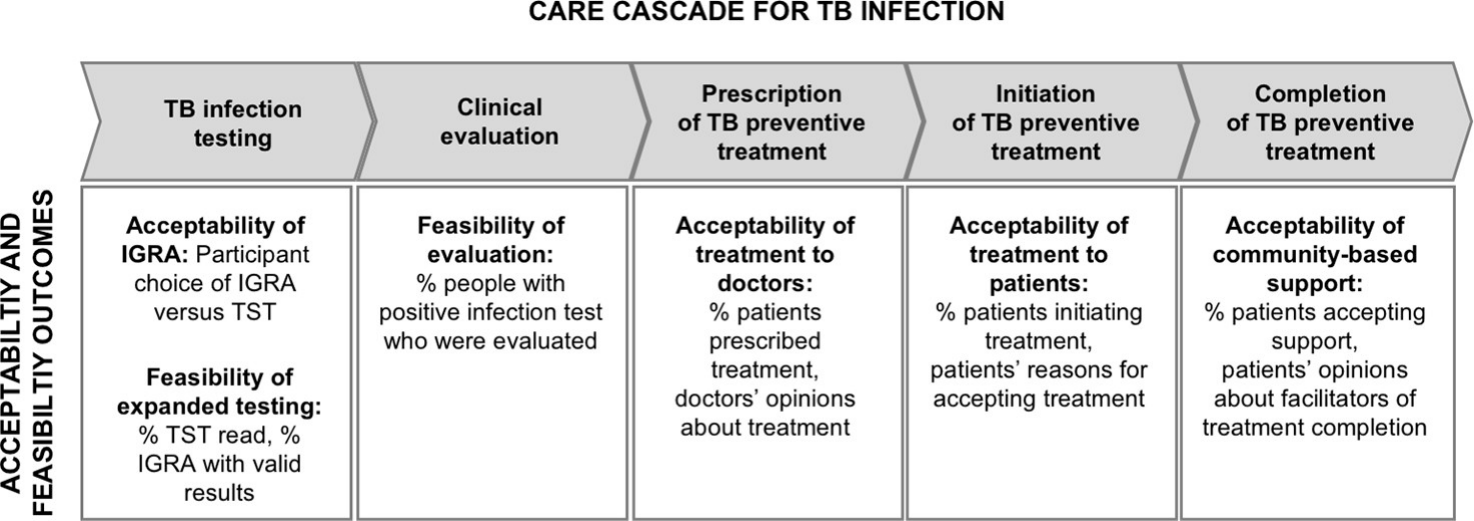
TB infection care cascade framework and key acceptability and feasibility outcomes of study. Abbreviations: TB = tuberculosis, IGRA = interferon gamma release assay, TST = tuberculin skin test.

We conducted a convergent mixed methods approach to evaluate key implementation outcomes of feasibility and acceptability [22], structuring the evaluation around a care cascade framework [23,24] (Fig 2). The quantitative evaluation focused on determining the percentage of participants completing each step of the care cascade among those eligible to complete it. The cumulative percentage completing the entire cascade was calculated by multiplying the percentages completing the individual steps [23]. To explain high or low completion of specific steps, we analyzed qualitative data from focus group discussions. We integrated quantitative and qualitative findings to draw conclusions about the acceptability of TB infection testing and preventive treatment, as well as the feasibility of IGRA testing and community-based treatment support.

#### Quantitative analysis

Study staff prospectively collected quantitative data on TB infection testing, which was managed by Socios En Salud, as well as participants’ preferences for treatment support (S2 Appendix) and self-reported adverse events (S1 Appendix). Quantitative data on other TB evaluation procedures, prescription, treatment initiation, and treatment completion were collected retrospectively from medical records.

To quantitatively evaluate the care cascade, we assessed completion of TB infection testing among enrolled participants; completion of TB evaluation (defined as a minimum of clinical examination and chest radiography) among those with a positive test for infection; prescription of preventive treatment among those potentially eligible for treatment; initiation of preventive treatment among those for whom treatment was prescribed; and completion of preventive treatment among those who initiated treatment. For the purposes of this evaluation, we used the following criteria to define potential eligibility for preventive treatment: (1) participants had a positive test for TB infection or were child contacts <5 years old, (2) participants underwent a clinical evaluation and had a chest radiograph, and were not diagnosed with active TB, and (3) for participants who were contacts, the patient to whom they had been exposed had not been diagnosed with drug-resistant TB.

Characteristics associated with completion of care cascade steps were evaluated statistically using a modified Poisson regression with robust error variances to estimate risk ratios adjusted for risk group, age group, and sex. We used age groups with policy or clinical relevance (e.g., children <5 have different eligibility criteria for treatment, age 35 is frequently used as a benchmark for increased risk of isoniazid hepatotoxicity [25]). The outcome of completing an infection test was assessed among all enrolled participants. The outcome of a positive TB infection test was assessed among all people with a test result. The outcome of completing evaluation was assessed among people <5 years old and those with a positive infection test. The outcome of initiating preventive treatment was evaluated among participants eligible for preventive treatment after evaluation, combining the cascade steps of prescription and initiation. The outcome of preventive treatment completion was evaluated among participants who initiated treatment. Statistical analyses were performed in SAS v9.4 (SAS Institute, Cary, NC).

#### Qualitative analysis

Qualitative data were collected through focus group discussions held as part of the planning process and during implementation to solicit feedback for intervention improvement (Table 1). These focus group discussions were meant to inform implementation and to provide preliminary insight into implementation barriers, which could later be explored through more rigorous methods. Overall, eight focus group discussions included 34 community participants and 5 health care worker participants; 30 (88%) participants were female. Focus group discussions were audio recorded and transcribed, and transcripts were uploaded into the qualitative analysis software Dedoose [26]. Coding followed a framework analysis approach [27] guided by the results of the quantitative analysis, and focused on identifying barriers and facilitators to completion of each cascade step. Qualitative methods adhered to the Consolidated Criteria for Reporting Qualitative Research (COREQ) [28]; details can be found in S3 Appendix.

**Table 1:** Description of focus group discussions.

Group	Project phase	Participant description	Number of participants	Topics discussed (duration)
1	Planning	Adults 18–45 years old from families affected by TB	5	What preventive treatment support strategies would be acceptable and effective in participants’ communities (30 minutes)
2	Planning	Adults 46–70 years old from families affected by TB	6	
3	Planning	Caregivers from families affected by TB	6	
4	Feedback	Adult contacts who completed treatment	5	Experiences during preventive treatment, factors that supported uptake and adherence, challenges faced, and ways in which future interventions could address these challenges (60 minutes)
5	Feedback	Adult contacts who did not complete treatment	2	
6	Feedback	Caregivers of child contacts who completed treatment	5	
7	Feedback	Caregivers of child contacts who did not complete treatment	5	
8	Feedback	Doctors (2 general, 1 pulmonologist) and nurses (2) who managed TB preventive treatment in public health facilities	5	Barriers and facilitators to TB infection management (90 minutes)

### Ethical considerations

This study was approved by the Ethics Committee of the Universidad Peruana Cayetano Heredia and the Institutional Review Board of Harvard Medical School. Verbal informed consent was obtained from all intervention participants, with parental consent for children <18 years old and assent from children 8–17 years old. Consent was obtained separately for the initial TB infection evaluation and for follow-up during TB infection treatment. Participants were free to decline evaluation procedures or intervention components without withdrawing from the study. A waiver of written informed consent was granted on the grounds that study activities constituted no more than minimal risk and that requiring written informed consent for routine procedures in a programmatic setting would not be feasible.

Verbal informed consent was obtained for all community focus group participants. A waiver of written informed consent was granted on the grounds that participation constituted no more than minimal risk and that complete confidentiality could best be assured in this way since a written consent form would be the only study document recording participants’ names. Written informed consent was obtained for health care worker focus group participants. Participants were provided transport (except for those participating in the videoconference discussion) and a gift card for the equivalent of 10 USD.

## Results

### TB preventive treatment care cascade completion

**Fig 3:**
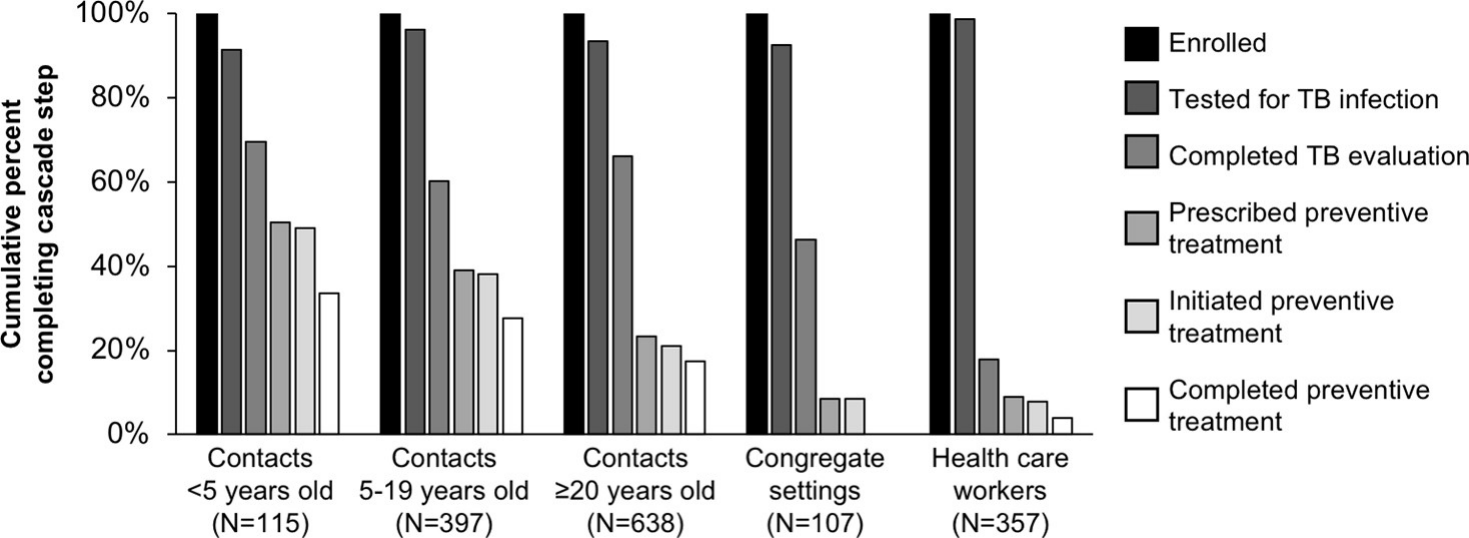
TB preventive treatment care cascade for contacts, people in congregate settings, and health care workers. Cumulative completion of each cascade step is calculated by calculating the percent who completed each step out of those eligible to complete it and then multiplying these percentages.

We enrolled 1,614 participants for TB infection evaluation: 1,002 household contacts, 148 non-household contacts, 107 residents and staff of congregate settings, and 357 health care workers. Females comprised 57% of household contacts, 63% of non-household contacts, 36% of congregate setting participants, and 73% of health care workers (S2 Table). Overall, cumulative completion of the TB preventive care cascade was 34% for contacts under 5 years old, 28% for contacts 5–19 years old, 18% for contacts at least 20 years old, 0% for people in congregate settings, and 4% for health care workers (Fig 3). The cascade step with the poorest completion varied among groups (S3 Table).

Overall, 1534 (95%) of enrolled participants had a TB infection test result, of whom 32% of household contacts, 18% of non-household contacts, 30% of health care workers, and 22% of people in congregate settings had a positive test result. The prevalence of infection increased with age group and was higher among contacts than other risk groups (Table 2). Overall, 59% of eligible participants completed evaluation, with health care workers significantly less likely to complete evaluation following a positive infection test than contacts (adjusted risk ratio [aRR] = 0.25, 95% confidence interval [CI] 0.16–0.38). Among participants eligible for preventive treatment after evaluation, adults were significantly less likely to initiate treatment than children (aRR for adults 20–35 years old = 0.56, 95% CI 0.39–0.82; aRR for adults >35 years old = 0.39, 95% CI 0.28–0.55). This gap was mostly attributable to doctors’ not prescribing preventive treatment rather than poor acceptance among participants, as only 49% of eligible participants were prescribed preventive treatment, while 95% of participants who were prescribed treatment initiated it (S3 Table). Overall, 72% of participants who initiated preventive treatment completed it, with no significant differences by age or sex.

**Table 2:** Predictors of TB infection, evaluation, preventive treatment initiation, and preventive treatment completion.

Participant characteristics		Enrolled	aRR (95% CI) for positive TB infection test	aRR (95% CI) for completing evaluation	aRR (95% CI) for initiating preventive treatment	aRR (95% CI) for completing preventive treatment
Risk group	Contacts	1,150	Reference	Reference	Reference	Not assessed
	Congregate setting	107	0.67 (0.48–0.93)	0.71 (0.46–1.10)	0.62 (0.18–2.17)	Not assessed
	Health care workers	357	0.73 (0.60–0.87)	0.25 (0.16–0.38)	1.37 (0.77–2.45)	Not assessed
Age group	0–4 years	115	Reference	Reference	Reference	Reference
	5–19 years	445	1.24 (0.74–2.08)	0.89 (0.72–1.10)	0.87 (0.66–1.16)	1.02 (0.79–1.32)
	20–35 years	424	2.28 (1.39–3.74)	0.93 (0.75–1.34)	0.56 (0.39–0.82)	1.00 (0.73–1.35)
	>35 years	630	3.49 (2.16–5.63)	1.06 (0.91–1.23)	0.39 (0.28–0.55)	1.06 (0.82–1.36)
Sex	Female	961	Reference	Reference	Reference	Reference
	Male	653	1.21 (1.04–1.41)	0.93 (0.92–1.06)	0.95 (0.75–1.19)	0.89 (0.72–1.09)

For the first three analyses, risk ratios are adjusted for risk group, age, and sex; for the analysis of predictors for completing preventive treatment, risk group is not assessed because of small numbers of non-contacts initiating treatment. Abbreviations: aRR = adjusted risk ratio, CI = confidence interval, TB = tuberculosis.

### Feasibility and acceptability indicators

#### TB infection testing

In total, 1,542 (96%) participants agreed to be tested for TB infection, of whom 1,080 (70%) had blood drawn for IGRA testing, and 465 (30%) had a TST placed; three individuals had both tests performed. In general, the participants followed age-based test type recommendations made by study staff, with 96% of tested children <10 years old having a TST placed, and 91% of tested adults (≥18 years old) having blood drawn for IGRA. However, among participants 10–17 years old, test choice was variable, with 52% undergoing IGRA and 48% TST. In total, 462 (99%) of the placed TSTs were read, and 1,075 (>99%) of the blood samples yielded non-indeterminate IGRA results.

#### Evaluation to diagnose TB infection and rule out TB disease

Clinical evaluation and chest radiography were completed for 80 (70%) children under 5 years old and for 245 (56%) older participants with a positive infection test (S3 Table), and 74% of the people who completed the evaluation also had sputum testing performed. Considering chest radiography, sputum testing, and clinical evaluation separately, the evaluation procedure completed by the fewest participants with TB infection was the clinical evaluation (70% of contacts, 23% of people in congregate settings, 50% of health care workers). In contrast, 90% of contacts, 44% of people in congregate settings, and 91% of health care workers completed at least one of the three evaluation procedures. TB disease was diagnosed for 15 individuals (12 household contacts, 2 non-household contacts, and 1 health care worker).

#### Preventive treatment prescription

Of the 281 participants eligible for preventive treatment after evaluation, only 139 (49%) were prescribed treatment. Prescription did not differ based on whether or not participants had abnormal chest radiographs (p = 0.744), whether or not they reported symptoms (p = 0.810). An additional 24 participants were prescribed treatment outside the guideline recommendations. These included participants with a negative infection test (n = 11), without an infection test (n = 3), without a chest radiograph (n = 2), and with exposure to a patient diagnosed with drug-resistant TB (n = 8). For this last group, we do not know whether the drug-resistant diagnosis was known at the time of prescribing preventive treatment.

#### Preventive treatment initiation and completion

Of 163 participants who were prescribed preventive treatment, 155 (95%) initiated treatment, and 112 (72%) of them completed treatment. Of the forms of treatment support offered to participants at the time of treatment initiation, 99% of participants accepted automated daily SMS reminders to take medications, 45% requested periodic reminder phone calls, and 60% requested periodic home visits from a community health worker. In addition, 19% requested another form of support, including visits to a location other than home or help with picking up medications.

Adverse events were reported by 25 (16%) participants during at least one monthly screening (S5 Table). These self-reported adverse events were more common for children <5 years old (16%) and adults >35 years old (32%) compared to children 5–19 years old (8%) and adults 20–35 years old (8%) (p = 0.222 for the comparison between the 5–19 and 0–4 age groups; p = 0.007 for the comparison between the 5–19 and >35 age groups). Two children and one adult >35 years old had treatment suspended for adverse events. No participants developed TB disease during treatment.

### Facilitators and barriers

Our focus group discussions focused on facilitators and barriers to prescription, initiation, and completion of treatment. Physicians and nurses identified several factors that prevent physicians from prescribing preventive treatment (Table 3). These included the lack of clear guidance for prescribing treatment for adult contacts in the national guidelines and a perception that preventive treatment is not for adults. They also mentioned that scientific knowledge about the efficacy or importance of preventive treatment is not widespread, and that some physicians believe that preventive treatment can cause drug resistance. A nurse mentioned that the pulmonologist visits to the health facilities were helpful for convincing doctors at the health facilities to prescribe treatment.

**Table 3:** Representative quotations illustrating facilitators and barriers to TB preventive treatment, by cascade step.

Cascade step	Theme	Participant, focus group	Example quotation
Prescription (facilitator)	Specialist consultations help convince doctors to prescribe	Nurse, group 8	We had the pulmonologist, who knows a lot. And it is good that he was there, because sometimes when our colleagues had doubts, we as nurses–because there is always more trust shown toward a doctor than a nurse–we could approach him.
Prescription (barrier)	Guidelines do not emphasize treatment	Pulmonologist, group 8	We are governed by a national guideline, and unfortunately, latent TB infection is given little importance in the national guideline; there are only a few paragraphs on latent TB infection.
	Lack of clear indications in guidelines	Nurse, group 8	What is not considered in the national guidelines is treatment for adults…it is not given, not considered for all adults. It would be good if they could come out with “positive PPD or IGRA” or some other indication.
	Lack of awareness of scientific evidence	Doctor, group 8	The doubts about effectiveness are widespread. In my opinion, I consider isoniazid to be a big help, that it could reduce TB incidence in Peru. But others always ask me about isoniazid or the evaluation of contacts or preventive treatment for contacts. . .People lack a lot of information, they may lack technical expertise, they lack awareness.
	Perception that treatment is not for adults	Pulmonologist, group 8	What happens is that the mentality of the majority of our colleagues is that [preventive] treatment is for children <5 years old and some people with risk factors.
	Concerns about drug resistance	Pulmonologist, group 8	There are many specialists who will argue that with the large amount of resistance we have, resistance to isoniazid in our setting, many colleagues argue that [preventive treatment] is not viable for this reason. But even if they are right, this primary resistance is not more than 10%, so there will still be benefit to preventive treatment.
Uptake (facilitator)	Counseling about rationale for treatment	Female, group 4	I was recently diagnosed with asthma…the doctor told me, “You are a person who uses corticosteroids, which means that you are a patient who is sensitive and at risk. Therefore, you should take [preventive treatment].”
	Fear of getting sick like family members	Female, group 7	When [my husband] coughed, he coughed up so much blood, almost half a bag of blood. Looking at him, I prayed to have strength for the sake of my child. So I started to take the [preventive treatment]…I saw [my husband] and said, “I do not want this to happen to me.”
Uptake (barrier)	Insurance status	Nurse, group 8	Here in Peru, we have two types of [public] insurance: the one from the Ministry of Health is SIS, which is what we work with, and EsSALUD [employer-based]. Many of our patients and families work and have other insurance, and sometimes these are the people who get [preventive] treatment prescribed. But because they have EsSALUD insurance, we cannot give them the isoniazid, and we cannot give it to their children. [EsSALUD hospitals] do not have isoniazid, so these patients or their family members come back and ask us to please give them [isoniazid], but it is complicated for us to give medications to a person who has a different insurance.
Completion (facilitator)	Family support	Female, group 4	At times I cannot go [to the health facility to pick up medications]. And my mother, because she is always going there, she picks them up for everyone and brings them to us.
	Community health workers offer support	Female, group 3	[Community health workers] have more training to be able to give you information. And if you have any doubts, you ask them, and they know how to respond. In contrast, a family member at times will tell you “fine, well, take [the medication] or don’t take it.”
	Personal strategies for remembering to take medications	Females, group 4	Participant 1: “I leave the pills on the table to remind myself” Participant 2: “I put them in my wallet.”
	SMS reminders help people remember to take medications	Female, group 2	They are hanging over their phones all day—they eat like that, have breakfast like that, with their phones. So when [the SMS] comes, with its notification tone, they remember.
Completion (barrier)	Medication fatigue and long treatment	Female, group 6	It has been very difficult for my daughter. She did not want to take [preventive treatment]. She took it for the first months. Then she got tired of it, she was sick of it and did not want to take it. She shouted and cried ‘I don’t want these pills.’
	Anticipated or experienced adverse events	Female, group 1	When my brother-in-law, when he takes [preventive treatment], he tells me that it gives him nausea, or maybe affects his liver. Well, I would like to take three months and no more.
	Inconvenience of weekly medication refills	Female, group 5	At times, we do not have the time to pick up the mediations weekly. There is no time to go and pick them up. In my case, I work in rotating shifts, and sometimes I do not have time to go….Could medication pickups be every two weeks instead? Weekly is very short.
	Difficulty giving pills to children	Female, group 3	My son suffers taking it. Although he chews it, the bitter flavor stays in his mouth, and he cries.
	Forgetting while busy	Female, group 5	Sometimes I would go to the health facility early with my daughter, or I would have something to do early, and I would come back late. And on these days I did not take the pills because I left the house very early and I left the pills at home.
	SMS reminders are not useful for everyone	Male, group 1	There are times when one may be sent a message–let’s say one sees it in the afternoon…but the person who saw it in the afternoon may continue their routine into the night and forget.

Additional supporting quotations can be found in S4 Table.

Regarding treatment initiation, contacts said that the counseling they received about the purpose of treatment was a motivating factor for accepting preventive treatment. Contacts also mentioned that they were motivated to accept treatment by the fear of getting sick like their family members with TB. A nurse mentioned that one barrier to treatment initiation was that contacts covered by employer-based health insurance could not receive medications from the public health facilities in their neighborhoods due to the separation of health services by insurance system.

In focus groups, participants described positive support from community health workers, support from family members, and personal strategies for reminding themselves to take medications as facilitators to adherence. While some participants felt that daily SMS reminders had been helpful, others did not. For example, one participant said that if a person receives an SMS reminder while he is out during the day, he might forget it by the time he gets home. The barriers to adherence identified by participants included medication fatigue, adverse events (both experienced and anticipated), the inconvenience of weekly medication refills, and difficulty administering pills to children.

## Discussion

This study represents an initial attempt to close the gap in TB preventive treatment in a middle-income setting by expanding preventive services to adult contacts and other risk groups. While completion of the TB preventive cascade was only 18–34% among contacts and poorer among health care workers and people in congregate settings, these results are consistent with what programs in high-income countries often achieve; a systematic review of the TB preventive care cascade, in which the majority of studies came from high-income countries, estimated cascade completion to be 19% globally [23]. Moreover, as a negligible number of adults in Peru currently access preventive treatment [18], this study represents an important initial step to expanding access. We found that TB infection testing and treatment were acceptable in this population, with >90% of participants completing testing and initiating treatment if prescribed. The biggest gaps in the care cascade were in completing evaluation following a positive test and having doctors prescribe preventive treatment to participants with TB infection [12].

Our intervention successfully expanded TB infection testing, with over 90% of all participant groups completing testing, and over 90% of adults accepting IGRA. From a staffing standpoint, using IGRA instead of TST made the intervention easier to implement because testing required only a single participant visit, and phlebotomy could be performed by technicians rather than nurses. However, our intervention benefitted from a donation of IGRA kits and a laboratory with experience performing IGRA. While studies from high-income countries have suggested that IGRA testing is more cost-effective than TST [29], either can be used to ensure that people with TB infection are identified for care.

The limited acceptability of preventive treatment among physicians is a major barrier to raising the standard of care, and to achieving global goals for TB elimination. Health care workers in our focus group suggested that clear messaging from the national or local TB program is necessary to address the perceived lack of prioritization of preventive treatment. However, the presence of strong recommendations is not sufficient for ensuring guideline compliance. TB preventive treatment for people living with HIV has been recommended by WHO since 2011 [8], and nearly all PEPFAR-supported countries incorporated this recommendation into their national guidelines. Yet, in a 2017 survey, only 60% of these countries reported nationwide implementation [30]. Ensuring that providers adopt evidence-based recommendations requires not only promoting awareness of national guidelines and international recommendations, but also targeted training, supervision, and infrastructure strengthening [31]. It will thus require significant effort to re-educate providers after decades of international health agency recommendations to focus TB preventive treatment only on child contacts <5 and people living with HIV—a standard of care that not only left many exposed and at-risk individuals untreated but was significantly different than practices that were propagated in wealthy countries [32].

In our study, completion of TB preventive treatment (72%) was relatively high compared to what has been reported in the literature [23,33], and adults were as likely to complete treatment as children. The fact that all participants accepted at least one option from an array of patient-centered supports suggests the importance of built-in programmatic flexibility. Overall, the adherence barriers that participants identified could be addressed through policy changes, without major infrastructural change. In health systems that use isoniazid-based preventive treatment, dispensing medications monthly and offering a child-friendly isoniazid formulation could help address the inconvenience of frequent pickups and the difficulty of administering pills to children. Other barriers such as medication fatigue and adverse events could be addressed by making newer, shorter regimens available. For example, a 4-month rifampicin regimen has fewer adverse events [34], and shortened regimens have better treatment completion [35–37].

We found health care workers and staff and residents of congregate settings were much less likely to complete the TB infection care cascade compared to contacts of patients with TB disease. However, our qualitative research was focused on testing and treatment for contacts, who comprised the majority of our target population. More work is required to understand the local barriers to TB infection testing and treatment for health care workers and people in congregate settings. Among health care workers in the United States, concern over adverse events and the stigma of TB preventive treatment are barriers to accepting TB treatment [38]. It is possible that similar concerns among Peruvian health care workers may explain why many were willing to be tested but few accepted treatment. Alternatively, lack of familiarity with IGRA among health workers could have also contributed to poor follow-up for positive results.

Strengths of our study include the structured implementation science approach and the integration of both quantitative and qualitative methods to evaluate acceptability and feasibility. This approach allowed us to both evaluate the performance of the intervention as a whole and understand how different elements of the intervention affected the feasibility of changing practice around TB infection testing and preventive treatment. This knowledge is important not only for program improvement in Peru, but also for highlighting programmatic considerations that may apply to other settings where TB preventive treatment has to date been restricted to young children and people living with HIV.

Our study had several important limitations related to the limited data collection that was feasible in the context of a programmatic intervention whose goal was to maximize reach. We did not collect comprehensive information from medical records, which would have been required to rigorously assess eligibility for TB infection treatment under national policy guidelines. Because we did not collect data on all the possible contraindications for TB preventive treatment, the number of participants we classified as eligible is a maximum estimate, and the percentage who were prescribed treatment is likely somewhat higher than what we have reported. Even so, prescription of TB preventive treatment to adult contacts was so low that the conclusion of suboptimal prescription is likely still valid.

Our study was also limited by the approach of using only focus group discussions held for intervention planning and feedback for our qualitative research. The number of groups was pre-determined by resource and time constraints. While certain themes (e.g., the importance of support from family and community health workers) arose repeatedly among different groups of contacts, our sample size was insufficient to achieve thematic saturation, and our sampling strategy prevented us from capturing the full diversity of relevant experiences. For example, because we recruited participants from families receiving preventive treatment, our insight into barriers to evaluation and treatment initiation was limited. Moreover, we held only one focus group discussion with health care workers and none with residents or staff of congregate settings. Finally, while we attempted to recruit both men and women for focus groups, most men refused because they had to work during the times that the focus groups were held; therefore, we were unable to gain much insight into the experiences of men or into how barriers may differ between men and women. For instance, working men might have identified barriers related to losing income in order to pick up medications or attend clinic appointments [39].

## Conclusions

In conclusion, expanding access to testing and treatment of TB infection in countries that have only prioritized children <5 and people living with HIV requires understanding facilitators and barriers to the care cascade. We observed high uptake of testing and completion of preventive treatment when we offered convenient TB infection testing and individualized community-based treatment support. However, completion of evaluation following a positive infection test and prescription of preventive treatment were suboptimal, suggesting the need for additional efforts to close these gaps. Solutions exist: patient incentives have been shown to be effective for increasing the completion of evaluation [40], while training, mentorship, and supervision for providers will be important to support the transition to best-practice care for TB infection [31]. These lessons can inform the efforts of coalitions seeking to implement a comprehensive approach to TB elimination that includes treatment of TB infection.

## Supporting information

S1 Table Barriers to TB preventive treatment previously identified in Peru and intervention components designed to address these barriers. (DOCX)

S2 Table Age and sex of enrolled participants, by risk group. (DOCX)

S3 Table Completion of TB infection treatment cascade steps, by risk group, age group, and sex. (DOCX)

S4 Table Supporting quotations relating to identified facilitators and barriers to TB preventive treatment, by cascade step. (DOCX)

S5 Table Self-reported adverse events among participants who initiated preventive treatment and completed at least one monthly adverse event screening, by age group. (DOCX)

S1 Appendix Data collection about adverse events. (DOCX)

S2 Appendix Data collection about support preferences. (DOCX)

S3 Appendix Qualitative research methods and adherence to COREQ checklist. (DOCX)

## References

[1] Keshavjee S, Dowdy D, Swaminathan S. Stopping the body count: a comprehensive approach to move towards zero tuberculosis deaths. Lancet. 2015;386(10010):e46–7. 10.1016/S0140-6736(15)00320-7 26515680

[2] Rangaka MX, Cavalcante SC, Marais BJ, Thim S, Martinson NA, Swaminathan S, et al Controlling the seedbeds of tuberculosis: diagnosis and treatment of tuberculosis infection. Lancet. 2015;386(10010):2344–53. 10.1016/S0140-6736(15)00323-2 26515679 PMC4684745

[3] Houben R, Menzies NA, Sumner T, Huynh GH, Arinaminpathy N, Goldhaber-Fiebert JD, et al Feasibility of achieving the 2025 WHO global tuberculosis targets in South Africa, China, and India: a combined analysis of 11 mathematical models. Lancet Glob Health. 2016;4(11):e806–e15. 10.1016/S2214-109X(16)30199-1 27720688 PMC6375908

[4] Targeted tuberculin testing and treatment of latent tuberculosis infection. American Thoracic Society. MMWR Recomm Rep. 2000;49(RR-6):1–51. 10881762

[5] Getahun H, Matteelli A, Abubakar I, Aziz MA, Baddeley A, Barreira D, et al Management of latent Mycobacterium tuberculosis infection: WHO guidelines for low tuberculosis burden countries. Eur Respir J. 2015;46(6):1563–76. 10.1183/13993003.01245-2015 26405286 PMC4664608

[6] McMillen CW. Discovering tuberculosis: a global history, 1900 to the present. New Haven and London: Yale University Press; 2015.

[7] World Health Organization. Recommendation for investigating contacts with infectious tuberculosis in low- and middle-income settings. Geneva: World Health Organization; 2012. 24404639

[8] World health Organization. Intensified tuberculosis case-finding and isoniazid preventive therapy for people living with HIV in resource-constrained settings. Geneva: World Health Organization; 2011.

[9] World Health Organization. Global Tuberculosis Report 2020. Geneva: World Health Organization; 2020.

[10] World Health Organization. Latent tuberculosis infection: updated and consolidated guidelines for programmatic management. Geneva: World Health Organization; 2018. 30277688

[11] Zero TB Initiative. Synthesis of guidelines on TB infection treatment: a tool for Zero TB coalitions. 2019. Available from: https://www.zerotbinitiative.org/resources-for-coalitions.

[12] United Nations high-level meeting on the fight against tuberculosis. Political declaration of the UN General Assembly high-level meeting. Resolution A/RES/73/3 adopted October 10, 2018. Available from: https://digitallibrary.un.org/record/1649568?ln=en.

[13] Cobelens F, van Kampen S, Ochodo E, Atun R, Lienhardt C. Research on implementation of interventions in tuberculosis control in low- and middle-income countries: a systematic review. PLoS Med. 2012;9(12):e1001358 10.1371/journal.pmed.1001358 23271959 PMC3525528

[14] Agins BD, Ikeda DJ, Reid MJA, Goosby E, Pai M, Cattamanchi A. Improving the cascade of global tuberculosis care: moving from the “what” to the “how” of quality improvement. Lancet Infect Dis. 2019;19(12):e437–e43. 10.1016/S1473-3099(19)30420-7 31447305

[15] Puma Abarca DV, Millones AK, Jimenez J, Brooks MB, Galea JT, Lecca L, et al Tuberculosis active case finding in high-burden areas: experience with mobile x-ray vans and Xpert MTB/RIF in Lima, Peru (OA-01-300-31). Int J Tuberc Lung Dis. 2019;22(11Suppl2):S63.

[16] Quispe N, Asencios L, Obregon C, Velasquez GE, Mitnick CD, Lindeborg M, et al The fourth national anti-tuberculosis drug resistance survey in Peru. Int J Tuberc Lung Dis. 2020;24(2):207–13. 10.5588/ijtld.19.0186 32127106 PMC7325691

[17] Ministerio de Salud. Norma técnica de salud para la atención integral de las personas afectadas por tuberculosis. Lima: Ministerio de Salud; 2013. Available from: http://www.tuberculosis.minsa.gob.pe/portaldpctb/recursos/20180308083418.pdf.

[18] Otero L, Battaglioli T, Rios J, De la Torre Z, Trocones N, Ordonez C, et al Contact evaluation and isoniazid preventive therapy among close and household contacts of tuberculosis patients in Lima, Peru: an analysis of routine data. Trop Med Int Health. 2020;25(3):346–56. 10.1111/tmi.13350 31758837 PMC7054138

[19] Yuen CM, Millones AK, Contreras CC, Lecca L, Becerra MC, Keshavjee S. Tuberculosis household accompaniment to improve the contact management cascade: A prospective cohort study. PLoS One. 2019;14(5):e0217104 10.1371/journal.pone.0217104 31100097 PMC6524822

[20] Chiang SS, Roche S, Contreras C, Del Castillo H, Canales P, Jimenez J, et al Barriers to the treatment of childhood tuberculous infection and tuberculosis disease: a qualitative study. Int J Tuberc Lung Dis. 2017;21(2):154–60. 10.5588/ijtld.16.0624 28234078

[21] Shu E, Sobieszczyk ME, Sal Y Rosas VG, Segura P, Galea JT, Lecca L, et al Knowledge of tuberculosis and vaccine trial preparedness in Lima, Peru. Int J Tuberc Lung Dis. 2017;21(12):1288–93. 10.5588/ijtld.17.0116 29297450

[22] Proctor E, Silmere H, Raghavan R, Hovmand P, Aarons G, Bunger A, et al Outcomes for implementation research: conceptual distinctions, measurement challenges, and research agenda. Adm Policy Ment Health. 2011;38(2):65–76. 10.1007/s10488-010-0319-7 20957426 PMC3068522

[23] Alsdurf H, Hill PC, Matteelli A, Getahun H, Menzies D. The cascade of care in diagnosis and treatment of latent tuberculosis infection: a systematic review and meta-analysis. Lancet Infect Dis. 2016;16(11):1269–78. 10.1016/S1473-3099(16)30216-X 27522233

[24] Zero TB Initiative. A best-practice framework of program indicators for monitoring a comprehensive approach to the tuberculosis epidemic. 2017. Available at: https://www.zerotbinitiative.org/resources-for-coalitions.

[25] Smith BM, Schwartzman K, Bartlett G, Menzies D. Adverse events associated with treatment of latent tuberculosis in the general population. CMAJ. 2011;183(3):E173–9. 10.1503/cmaj.091824 21220436 PMC3042475

[26] Dedoose Version 8.0.35, web application for managing, analyzing, and presenting qualitative and mixed method research data. 2018 Los Angeles, CA: SocioCultural Research Consultants LLC.

[27] Smith J, Firth J. Qualitative data analysis: the framework approach. Nurs Res. 2011;18(2):52–62. 10.7748/nr2011.01.18.2.52.c8284 21319484

[28] Tong A, Sainsbury P, Craig J. Consolidated criteria for reporting qualitative research (COREQ): a 32-item checklist for interviews and focus groups. Int J Qual Health Care. 2007;19(6):349–57. 10.1093/intqhc/mzm042 17872937

[29] Nienhaus A, Schablon A, Costa JT, Diel R. Systematic review of cost and cost-effectiveness of different TB-screening strategies. BMC Health Serv Res. 2011;11:247 10.1186/1472-6963-11-247 21961888 PMC3196701

[30] Surie D, Interrante JD, Pathmanathan I, Patel MR, Anyalechi G, Cavanaugh JS, et al Policies, practices and barriers to implementing tuberculosis preventive treatment-35 countries, 2017. Int J Tuberc Lung Dis. 2019;23(12):1308–13. 10.5588/ijtld.19.0018 31931915

[31] Rowe AK, Rowe SY, Peters DH, Holloway KA, Chalker J, Ross-Degnan D. Effectiveness of strategies to improve health-care provider practices in low-income and middle-income countries: a systematic review. Lancet Glob Health. 2018;6(11):e1163–e75. 10.1016/S2214-109X(18)30398-X 30309799 PMC6185992

[32] World Health Organization. Guidelines on the management of latent tuberculosis infection. Geneva: World Health Organization; 2015. 25973515

[33] Szkwarko D, Hirsch-Moverman Y, Du Plessis L, Du Preez K, Carr C, Mandalakas AM. Child contact management in high tuberculosis burden countries: A mixed-methods systematic review. PLoS One. 2017;12(8):e0182185 10.1371/journal.pone.0182185 28763500 PMC5538653

[34] Campbell JR, Trajman A, Cook VJ, Johnston JC, Adjobimey M, Ruslami R, et al Adverse events in adults with latent tuberculosis infection receiving daily rifampicin or isoniazid: post-hoc safety analysis of two randomised controlled trials. Lancet Infect Dis. 2020;20(3):318–29. 10.1016/S1473-3099(19)30575-4 31866327

[35] Macaraig MM, Jalees M, Lam C, Burzynski J. Improved treatment completion with shorter treatment regimens for latent tuberculous infection. Int J Tuberc Lung Dis. 2018;22(11):1344–9. 10.5588/ijtld.18.0035 30355415 PMC6309173

[36] Cruz AT, Starke JR. Completion Rate and Safety of Tuberculosis Infection Treatment With Shorter Regimens. Pediatrics. 2018;141(2). 10.1542/peds.2017-2838 29363561

[37] Ronald LA, FitzGerald JM, Bartlett-Esquilant G, Schwartzman K, Benedetti A, Boivin JF, et al Treatment with isoniazid or rifampin for latent tuberculosis infection: population-based study of hepatotoxicity, completion and costs. Eur Respir J. 2020;55(3). 10.1183/13993003.02048-2019 31980498

[38] Joseph HA, Shrestha-Kuwahara R, Lowry D, Lambert LA, Panlilio AL, Raucher BG, et al Factors influencing health care workers’ adherence to work site tuberculosis screening and treatment policies. Am J Infect Control. 2004;32(8):456–61. 10.1016/j.ajic.2004.06.004 15573052

[39] Jacobson KB, Niccolai L, Mtungwa N, Moll AP, Shenoi SV. “It’s about my life”: facilitators of and barriers to isoniazid preventive therapy completion among people living with HIV in rural South Africa. AIDS Care. 2017;29(7):936–42. 10.1080/09540121.2017.1283390 28147705 PMC5545149

[40] Barss L, Moayedi-Nia S, Campbell JR, Oxlade O, Menzies D. Interventions to reduce losses in the cascade of care for latent tuberculosis: a systematic review and meta-analysis. Int J Tuberc Lung Dis. 2020;24(1):100–9. 10.5588/ijtld.19.0185 32005312

