# Supplementary material for: Closing delivery gaps in the treatment of tuberculosis infection: Lessons from implementation research in Peru
Source: PLoS One. 2021 Feb 19;16(2):e0247411. doi: 10.1371/journal.pone.0247411 (PMC7895363; doi:10.1371/journal.pone.0247411)
Supplement: S1 Table — (DOCX) [file pone.0247411.s001.docx]

**Table S1. Barriers to TB preventive treatment previously identified in Peru, and intervention components designed to address these barriers**

| **TB infection cascade step** | **Barriers previously identified** | **Components to address barriers** |
| --- | --- | --- |
| Testing for TB infection | - Difficulty coordinating appointments for tuberculin skin test placement and reading at health facilities - Concerns about conserving limited tuberculin supply | - Intervention offered interferon gamma release assay tests with phlebotomy performed at convenient locations and times |
| Evaluation for TB disease | - Patients need help navigating local health services to complete evaluation - Providers refer to regional hospitals when they do not feel confident ruling out TB, and these referrals are difficult for patients to complete | - Field team helped coordinate appointments for evaluation procedures - Chest radiography provided in an x-ray van at community locations - Need for hospital referrals reduced by deploying specialists to regularly visit primary care facilities to provide consultation |
| Prescription of TB preventive treatment | - Providers do not prescribe preventive treatment to all contacts who should receive treatment according to the National Guidelines - Providers are skeptical of the importance of preventive treatment - Providers fear that that preventive treatment generates drug resistance | - Specialists who visited primary care facilities provided information about preventive treatment and offered guidance during TB evaluation |
| TB preventive treatment initiation | - Close contacts of TB patients have low knowledge about risk of TB - However, uptake of preventive treatment is high | - Field team provided counseling about TB infection and its treatment before testing and during treatment |
| TB preventive treatment completion | - People get tired of taking a long treatment and can forget to take medications - Directly observed therapy is labor-intensive and not acceptable to all families; however, families receiving directly observed therapy appreciated not having to pick up medications themselves | - Automated daily SMS reminders to take medications; periodic SMS reminders to call a field team member if adverse events developed - Periodic phone calls and visits from community health workers tailored to patient preferences - Coordination of biweekly rather than weekly medication refills for participants struggling to access health facilities weekly |
