# Supplementary material for: Closing delivery gaps in the treatment of tuberculosis infection: Lessons from implementation research in Peru
Source: PLoS One. 2021 Feb 19;16(2):e0247411. doi: 10.1371/journal.pone.0247411 (PMC7895363; doi:10.1371/journal.pone.0247411)
Supplement: S2 Table — (DOCX) [file pone.0247411.s002.docx]

**Table S2.** **Age and sex of enrolled participants, by risk group**

| **Participant characeristics** | | **Household contacts (N=1,002)** | **Non-household close contacts (N=148)** | **Congregate setting residents and staff (N=107)** | **Health care workers (N=357)** |
| --- | --- | --- | --- | --- | --- |
| Age group | 0-4 years | 99 (10) | 16 (11) | 0 (0) | 0 (0) |
|  | 5-19 years | 333 (33) | 64 (43) | 48 (45) | 0 (0) |
|  | 20-35 years | 248 (25) | 25 (17) | 15 (14) | 136 (38) |
|  | >35 years | 322 (32) | 43 (29) | 44 (41) | 221 (62) |
| Sex | Female | 570 (57) | 93 (63) | 39 (36) | 259 (73) |
|  | Male | 432 (43) | 55 (37) | 68 (64) | 98 (27) |

Column percentages are presented in parentheses.
