# Supplementary material for: Closing delivery gaps in the treatment of tuberculosis infection: Lessons from implementation research in Peru
Source: PLoS One. 2021 Feb 19;16(2):e0247411. doi: 10.1371/journal.pone.0247411 (PMC7895363; doi:10.1371/journal.pone.0247411)
Supplement: S3 Table — (DOCX) [file pone.0247411.s003.docx]

**Table S3.** **Completion of TB infection treatment cascade steps, by risk group, age group, and sex**

| **Participant characteristics** | | **Completed TB infection test^a^** | **Positive TB infection test^b^** | **Completed TB infection evaluation^c^** | **Prescribed preventive treatment^d^** | **Initiated preventive treatment^e^** | **Completed preventive treatment^f^** |
| --- | --- | --- | --- | --- | --- | --- | --- |
| Risk group | Contacts | 1,083 / 1,150 (94) | 327 / 1,083 (30) | 295 / 427  (69) | 128 / 252  (51) | 145 / 152  (95) | 108 / 145 (74) |
|  | Congregate setting | 99 / 107  (93) | 22 / 99  (22) | 11 / 11  (50) | 2 / 11  (18) | 2 / 2  (100) | 0 / 2  (0) |
|  | Health care workers | 352 / 357  (99) | 105 / 352  (30) | 18 / 105  (18) | 9 / 18  (50) | 8 / 9  (89) | 4 / 8  (50) |
| Age group | 0-4 years | 105 / 115  (91) | 15 / 105  (14) | 80 / 115  (70) | 53 / 73  (73) | 57 / 58  (98) | 40 / 57  (70) |
|  | 5-19 years | 428 / 445  (96) | 72 / 428  (17) | 45 / 72  (63) | 26 / 40  (65) | 40 / 41  (98) | 29 / 40  (73) |
|  | 20-35 years | 403 / 424  (95) | 114 / 403  (28) | 59 / 114  (52) | 22 / 51  (43) | 24 / 25  (96) | 17 / 24  (71) |
|  | >35 years | 598 / 630  (95) | 253 / 589  (42) | 141 / 253  (56) | 38 / 117  (32) | 34 / 39  (87) | 26 / 34  (76) |
| Sex | Female | 919 / 961  (96) | 258 / 919  (28) | 181 / 309  (59) | 81 / 155  (52) | 88 / 95  (93) | 67 / 88  (76) |
|  | Male | 615 / 653  (94) | 196 / 615  (32) | 144 / 245  (59) | 58 / 126  (46) | 67 / 68  (99) | 45 / 67  (67) |
| Total | | 1,534 / 1,614  (95) | 454 / 1,534  (30) | 325 / 554  (59) | 139 / 281  (49) | 155 / 163  (95) | 112/155  (72) |

Each cell shows the number who completed the cascade step / the number eligible to complete that step, with the percentage completing out of those eligible in parentheses. Definitions of eligibility are shown below.

^a^ All who were enrolled were eligible to complete a TB infection test. A complete test means that the tuberculin skin test (TST) was read or that the interferon gamma release assay (IGRA) yielded a non-indeterminate result.

**^b^** All people with a completed test were eligible to have a positive result.

^c^ For children 0-4 years old, all enrolled children were eligible to complete evaluation because isoniazid preventive treatment is indicated regardless of the infection test result; for all other age groups, only those with a positive infection test result were eligible to complete evaluation.

^d^ People were considered eligible to be prescribed preventive treatment if they met the following criteria: (1) participants had a positive test for TB infection or were child contacts <5 years old, (2) participants underwent a clinical evaluation and had a chest radiograph, and were not diagnosed with active TB, and (3) for participants who were contacts, the patient to whom they had been exposed had not been diagnosed with drug-resistant TB.

^e^ People who were prescribed preventive treatment were eligible to initiate preventive treatment. This includes 24 people who did not meet the eligibility criteria for preventive treatment but were prescribed treatment anyway.

^f^ People who initiated preventive treatment were eligible to complete preventive treatment.
