# Supplementary material for: Closing delivery gaps in the treatment of tuberculosis infection: Lessons from implementation research in Peru
Source: PLoS One. 2021 Feb 19;16(2):e0247411. doi: 10.1371/journal.pone.0247411 (PMC7895363; doi:10.1371/journal.pone.0247411)
Supplement: S4 Table — (DOCX) [file pone.0247411.s004.docx]

**Table S4. Supporting quotations relating to identified facilitators and barriers to TB preventive treatment, by cascade step**

| **Cascade step** | **Theme** | **Participant, focus group** | **Example quotation** |
| --- | --- | --- | --- |
| Prescription (facilitator) | Specialist consultations help convince doctors to prescribe | Nurse,  group 8 | We had the pulmonologist, who knows a lot. And it is good that he was there, because sometimes when our colleagues had doubts, we as nurses – because there is always more trust shown toward a doctor than a nurse – we could approach him. |
| Prescription (barrier) | Guidelines do not emphasize treatment | Pulmonologist, group 8 | We are governed by a national guideline, and unfortunately, latent TB infection is given little importance in the national guideline; there are only a few paragraphs on latent TB infection. |
|  | Lack of clear indications in guidelines | Nurse,  group 8 | What is not considered in the national guidelines is treatment for adults…it is not given, not considered for all adults. It would be good if they could come out with “positive PPD or IGRA” or some other indication. |
|  |  | Pulmonologist, group 8 | There is a lack of criteria because every document, whether it be national or international, has a certain flexibility….[the national guideline] says that one may prescribe [preventive treatment] to adults after medical evaluation; that is, the indication exists, there is flexibility in the document. But at the administrative level – for example in the pharmacy – this does not count for them. |
|  | Lack of awareness of scientific evidence | Doctor,  group 8 | The doubts about effectiveness are widespread. In my opinion, I consider isoniazid to be a big help, that it could reduce TB incidence in Peru. But others always ask me about isoniazid or the evaluation of contacts or preventive treatment for contacts...People lack a lot of information, they may lack technical expertise, they lack awareness. |
|  |  | Nurse,  group 8 | I believe that there is no better thing than for health care workers to see the evidence that this really does work….Many health care workers do not manage [TB infection] much. |
|  | Perception that treatment is not for adults | Pulmonologist, group 8 | What happens is that the mentality of the majority of our colleagues is that treatment is for children <5 years old and some people with risk factors. |
|  |  | Doctor,  group 8 | There was some doubt among the doctors about whether to give preventive treatment to patients with a positive IGRA, but who were older – for example, 40 or 50 years old. |
|  | Concerns about drug resistance | Pulmonologist, group 8 | There are many specialists who will argue that with the large amount of resistance we have, resistance to isoniazid in our setting, many colleagues argue that [preventive treatment] is not viable for this reason. But even if they are right, this primary resistance is not more than 10%, so there will still be benefit to preventive treatment. |
|  |  | Nurse,  group 8 | One [barrier] is the fear that when the medication is taken by health care workers, that the medication can cause resistance. |
| Uptake (facilitator) | Counseling about rationale for treatment | Female, group 4 | I was recently diagnosed with asthma…the doctor told me, “You are a person who uses corticosteroids, which means that you are a patient who is sensitive and at risk. Therefore, you should take [preventive treatment].” |
|  |  | Female, group 4 | Facilitator: Why did you decide to accept preventive treatment? Participant: Above all, it was because it is something good for us, to avoid things. At times we do not know what could happen to our health. Facilitator: Did they explain to you why you were going to receive this treatment? Participant: Yes, yes, they explained - they told me it is to protect me. |
|  | Fear of getting sick like family members | Female, group 5 | Facilitator: What was your motive for accepting preventive treatment? Participant: More than anything for prevention since we have my father who is sick; to avoid contagion or consequences in the future. |
|  |  | Female, group 7 | When [my husband] coughed, he coughed up so much blood, almost half a bag of blood. Looking at him, I prayed to have strength for the sake of my child. So I started to take the [preventive treatment]…I saw [my husband] and said, “I do not want this to happen to me.” |
| Uptake (barrier) | Insurance status | Nurse,  group 8 | Here in Peru, we have two types of [public] insurance: the one from the Ministry of Health is SIS, which is what we work with, and ESSALUD [employer-based]. Many of our patients and families work and have other insurance, and sometimes these are the people who get [preventive] treatment prescribed. But because they have ESSALUD insurance, we cannot give them the isoniazid, and we cannot give it to their children. [ESSALUD hospitals] do not have isoniazid, so these patients or their family members come back and ask us to please give them [isoniazid], but it is complicated for us to give medications to a person who has a different insurance. |
| Completion (facilitator) | Family support | Female, group 4 | At times I cannot go [to the health facility to pick up medications]. And my mother, because she is always going there, she picks them up for everyone and brings them to us. |
|  |  | Female, group 4 | My daughter, since she is receiving treatment, picks up the medications for both of us. |
|  |  | Female, group 6 | My son, who is 7 years old, he would remember after eating, “Mama, I get my pill.” “Yes, take it,” I say to him. At times I forget, and he says, “Mama, take your pill.” |
|  | Community health workers offer support | Male,  group 3 | At times, your family can say, “oh, just leave it” or “do it later.” But when [the community health workers] visit, they tell me to take [the medications], ask if I am taking them; and sometimes they call to ask if I am taking them, and make me remember. |
|  |  | Female, group 3 | [Community health workers] have more training to be able to give you information. And if you have any doubts, you ask them, and they know how to respond. In contrast, a family member at times will tell you “fine, well, take [the medication] or don’t take it.” |
|  |  | Female, group 6 | God bless these people who take their work so seriously. They take everything seriously and make us feel like we were part of their family. My husband and I are very thankful for this. |
|  |  | Female, group 4 | I feel very good because at least there is someone who is worrying about me. |
|  | Personal strategies for remembering to take medications | Females, group 4 | Participant 1: “I leave the pills on the table to remind myself” Participant 2: “I put them in my wallet.” |
|  |  | Female, group 4 | I have a piece of paper where I put the name of each of the boys who has to take the medications. They have to take the medications, and ours - for adults - is separate. So I put three containers on the table, with their names. |
|  | SMS reminders help people remember to take medications | Female, group 2 | They are hanging over their phones all day - they eat like that, have breakfast like that, with their phones. So when [the SMS] comes, with its notification tone, they remember. |
|  |  | Female, group 6 | Sometimes, when I am at the point of giving the medication to him, at times I forget. And [the SMS] comes and I give the medication to my son. |
| Completion (barrier) | Medication fatigue and long treatment | Female, group 6 | It has been very difficult for my daughter. She did not want to take [preventive treatment]. She took it for the first months. Then she got tired of it, she was sick of it and did not want to take it. She shouted and cried ‘I don’t want these pills.’ |
|  |  | Female, group 6 | I would like the pills to be not in the doses that they are now, but something not taken daily. Because [the children] get fed up with it…if it were two times a week, I think that would be more tolerable for them to take their pills or syrups. |
|  | Anticipated or experienced adverse events | Female, group 1 | When my brother-in-law, when he takes [preventive treatment], he tells me that it gives him nausea, or maybe affects his liver. Well, I would like to take three months and no more. |
|  |  | Female, group 4 | Facilitator: You were afraid [treatment] would give you gastritis? Participant: No, I already suffer from gastritis. Facilitator: Ah, you already have gastritis. Participant: Yes. Facilitator: And you thought this would make it worse? Participant: Exactly. I was worried because I was taking three pills every day. |
|  |  | Female, group 7 | My daughter said ‘Mama, no, it is going to upset her stomach.’ That is what she said, and that is what she still says. The [community health worker] told us, ‘no, it is not going to do anything to her.’…but you know what they say – where the captain rules, the sailor has no power. |
|  |  | Female, group 7 | They stopped treatment because he developed an allergy – like little blisters, he scratched and scratched and scratched. And when he stopped, it went back to normal. |
|  | Inconvenience of frequent medication refills | Female, group 5 | My father could not get up because he was bedridden, then I had to pick up the medications…The people from SES called me or wrote to me, saying ‘why are you not coming to pick up your medicine?’ Look, what happened is that I did not have time. |
|  |  | Female, group 5 | At times, we do not have the time to pick up the mediations weekly. There is no time to go and pick them up. In my case, I work in rotating shifts, and sometimes I do not have time to go….Could medication pickups be every two weeks instead? Weekly is very short. |
|  | Difficulty giving pills to children | Female, group 3 | My son suffers taking it. Although he chews it, the bitter flavor stays in his mouth, and he cries. |
|  | Forgetting while busy | Female, group 5 | Sometimes I would go to the health facility early with my daughter, or I would have something to do early, and I would come back late. And on these days I did not take the pills because I left the house very early and I left the pills at home. |
|  | SMS reminders are not useful for everyone | Male,  group 1 | There are times when one may be sent a message – let’s say one sees it in the afternoon…but the person who saw it in the afternoon may continue their routine into the night and forget. |
|  |  | Female, group 6 | They told me they would send a message, but the messages never came. |
