# Supplementary material for: Closing delivery gaps in the treatment of tuberculosis infection: Lessons from implementation research in Peru
Source: PLoS One. 2021 Feb 19;16(2):e0247411. doi: 10.1371/journal.pone.0247411 (PMC7895363; doi:10.1371/journal.pone.0247411)
Supplement: S5 Table — (DOCX) [file pone.0247411.s005.docx]

**Table S5. Self-reported adverse events among participants who initiated preventive treatment and completed at least one monthly adverse event screening, by age group**

| **Adverse event reported during verbal screening questionnaire** | **Number of patients reporting adverse event at ≥1 monthly screening (%)** | | | | |
| --- | --- | --- | --- | --- | --- |
|  | **0-4 years old (N=57)** | **5-19 years old (N=40)** | **20-35 years old (N=24)** | **≥36 years old (N=34)** | **Total**  **(N=155)** |
| Nausea | 0 (0) | 0 (0) | 1 (4) | 1 (3) | 2 (1) |
| Vomiting | 0 (0) | 0 (0) | 1 (4) | 2 (6) | 3 (2) |
| Loss of appetite | 3 (5) | 1 (3) | 1 (4) | 0 (0) | 5 (3) |
| Stomach ache | 3 (5) | 2 (5) | 0 (0) | 6 (18) | 11 (7) |
| Trouble sleeping | 0 (0) | 0 (0) | 1 (4) | 1 (3) | 2 (1) |
| Dark urine | 0 (0) | 0 (0) | 0 (0) | 2 (6) | 2 (1) |
| Yellow color in skin or eyes | 0 (0) | 0 (0) | 0 (0) | 0 (0) | 0 (0) |
| Skin rash | 2 (4) | 0 (0) | 0 (0) | 0 (0) | 2 (1) |
| Numbness or tingling in fingers or toes | 1 (2) | 0 (0) | 0 (0) | 3 (9) | 4 (3) |
| Fatigue or malaise | 2 (4) | 2 (5) | 0 (0) | 5 (15) | 9 (6) |
| Any other symptom that was concerning to the participant | 2 (4) | 1 (3) | 0 (0) | 1 (3) | 4 (3) |
| **Any of the above*** | 9 (16) | 3 (8) | 2 (8) | 11 (32) | 25 (16) |

*Since each participant could report multiple adverse events, the number of participants reporting any adverse event is not equal to the sum of the numbers of patients reporting each type of adverse event
