# Supplementary material for: Closing delivery gaps in the treatment of tuberculosis infection: Lessons from implementation research in Peru
Source: PLoS One. 2021 Feb 19;16(2):e0247411. doi: 10.1371/journal.pone.0247411 (PMC7895363; doi:10.1371/journal.pone.0247411)
Supplement: S1 Appendix — (DOCX) [file pone.0247411.s006.docx]

**Appendix S1**: **Data collection about adverse events**

This questionnaire was previously developed for a household contact management intervention in 2015^[[1]](#footnote-1)^ and comprised a Spanish translation of a standard isoniazid preventive side effect treatment monitoring form used in the United States. It was administered monthly either by phone or in person during a home visit to each person receiving preventive treatment.

***English***

|  | **Question** | **Response** |
| --- | --- | --- |
| 1 | Have you had nausea in the last month? | __ No __ Just once __ Repeatedly |
| 2 | Have you had vomiting in the last month? | __ No __ Just once __ Repeatedly |
| 3 | Have you had loss of appetite in the last month? | __ No __ Just once __ Repeatedly |
| 4 | Have you had stomach pain in the last month? | __ No __ Just once __ Repeatedly |
| 5 | Have you had insomnia in the last month? | __ No __ Just once __ Repeatedly |
| 6 | Have you had dark urine in the last month? | __ No __ Just once __ Repeatedly |
| 7 | Have you experienced yellowing of the skin or eyes in the last month? | __ No __ Just once __ Repeatedly |
| 8 | Have you had a rash in the last month? | __ No __ Just once __ Repeatedly |
| 9 | Have you had numbness or tingling in the fingers or toes in the last month? | __ No __ Just once __ Repeatedly |
| 10 | Have you had fatigue or malaise in the last month? | __ No __ Just once __ Repeatedly |
| 11 | Have you had any other discomfort in the past month? | __ No __ Just once __ Repeatedly  Describe**:** |

***Spanish***

|  | **Pregunta** | **Respuesta** |
| --- | --- | --- |
| 1 | ¿Ha tenido nauseas en el último mes? | __ No __ Solo una vez __ Sucedió repetidamente |
| 2 | ¿Ha tenido vómitos en el último mes? | __ No __ Solo una vez __ Sucedió repetidamente |
| 3 | ¿Ha tenido pérdida de apetito en el último mes? | __ No __ Solo una vez __ Sucedió repetidamente |
| 4 | ¿Ha tenido dolor de estómago en el último mes? | __ No __ Solo una vez __ Sucedió repetidamente |
| 5 | ¿Ha presentado insomnio en el último mes? | __ No __ Solo una vez __ Sucedió repetidamente |
| 6 | ¿Ha observado su orina oscura en el último mes? | __ No __ Solo una vez __ Sucedió repetidamente |
| 7 | ¿Ha presentado coloración amarillenta de la piel u ojos en el último mes? | __ No __ Solo una vez __ Sucedió repetidamente |
| 8 | ¿Ha presentado erupción cutánea en el último mes? | __ No __ Solo una vez __ Sucedió repetidamente |
| 9 | ¿Ha presentado entumecimiento u hormigueo en los dedos de las manos o pies en el último mes? | __ No __ Solo una vez __ Sucedió repetidamente |
| 10 | ¿Ha presentado fatiga o malestar en el último mes? | __ No __ Solo una vez __ Sucedió repetidamente |
| 11 | ¿Ha presentado alguna otra molestia en el último mes? | __ No __ Solo una vez __ Sucedió repetidamente  Describe**:** |

1. Yuen CM, Millones AK, Contreras CC, Lecca L, Becerra MC, Keshavjee S. Tuberculosis household accompaniment to improve the contact management cascade: a prospective cohort study. *PLOS One*. 2019; 14(5):e0217104 [↑](#footnote-ref-1)
