# Supplementary material for: Closing delivery gaps in the treatment of tuberculosis infection: Lessons from implementation research in Peru
Source: PLoS One. 2021 Feb 19;16(2):e0247411. doi: 10.1371/journal.pone.0247411 (PMC7895363; doi:10.1371/journal.pone.0247411)
Supplement: S2 Appendix — (DOCX) [file pone.0247411.s007.docx]

**Appendix S2. Data collection about support preferences**

For each participant who initiated preventive treatment, the field team offered (verbally) a standard menu of treatment support options. Participants could indicate multiple forms of treatment support. A standardized form (shown below) was used to register each participant’s preferences for treatment support so that the field team could provide support according to the participant’s stated preference. As the purpose of this questionnaire was programmatic case management, it was not tested or validated as a research instrument.

***English***

|  | **Question** | **Response** |
| --- | --- | --- |
| 1 | Method of treatment support | __ Phone calls (to participant) + SMS  __ Phone calls to a family member + SMS  __ Home visits + SMS  __ No support  __ Other: ______________ |
| 2 | Frequency of calls or visit | __ Weekly  __ Every 2 weeks  __ Monthly  __ Other: _______________ |

***Spanish***

|  | **Pregunta** | **Respuesta** |
| --- | --- | --- |
| 1 | Método de apoyo durante el tratamiento | __ Llamadas (al participante) + mensajes  __ Llamadas a un familiar + mensajes  __ Visitas domiciliarias + mensajes  __ No método de apoyo  __ Otro: ________________ |
| 2 | Frecuencia de llamadas o visitas | __ Semanal  __ Quincenal  __ Mensual  __ Otro: ________________ |
