# Supplementary material for: Closing delivery gaps in the treatment of tuberculosis infection: Lessons from implementation research in Peru
Source: PLoS One. 2021 Feb 19;16(2):e0247411. doi: 10.1371/journal.pone.0247411 (PMC7895363; doi:10.1371/journal.pone.0247411)
Supplement: S3 Appendix — (DOCX) [file pone.0247411.s008.docx]

**Appendix S3. Qualitative research methods and adherence to COREQ checklist**

The methods below are grouped by based on the items in the Consolidated Criteria for Reporting Qualitative Research (COREQ) checklist.

*Research team experience (COREQ items 1-5)*

Focus group discussions (FGDs) 1-7 were facilitated by a female Peruvian nurse (AKM), who was the study coordinator. FGD 1 was co-facilitated by JTG, an experienced qualitative researcher whose research has mostly been done in Peru. FGD 8 was co-facilitated by the study coordinator (AKM); the lead author (CMY), a female epidemiologist with experience working with the Peruvian health system; and a male US-based infectious disease doctor with experience managing TB infection treatment, as well as experience working with the Peruvian health system (DBT, listed in acknowledgements). Facilitators were advised in FGD facilitation by JTG, an experienced qualitative researcher, who also helped to develop the FGD guides.

*Relationships between facilitators and participants (COREQ items 6-8)*

Facilitators introduced themselves and their professions. In general, participants in FGD 1-7 did not know the facilitator (AKM), but a few had previously met her years ago when she was the coordinator of a program providing support to families affected by TB. The health care workers in FGD 8 all knew the study coordinator (AKM), and the doctors had previously met the lead author (CMY) and the US-based infectious disease doctor (DBT).

*Methodological orientation and participant selection (COREQ items 9-13)*

We used a Framework Analysis approach based on the latent TB infection cascade of care. The number of FGD discussions and the target of 5 participants for each group was pre-determined based on time and resource constraints.

Initially, the cascade step that most concerned us in terms of program planning was treatment completion, based on prior experience from Peru and published in the literature from other settings. We therefore focused our planning FGD (groups 1-3) on different age groups who might require different forms of support. Participants for these FGD were purposively sampled from families with a member currently receiving treatment for TB, thus representing the target population for preventive treatment. While all of these families had at least one family member receiving preventive treatment, we did not specifically recruit for the focus group participants who were themselves receiving preventive treatment. In our patient feedback FGD (groups 4-7), we sought to capture a diversity of experiences, including people who completed treatment and people who did not complete treatment. All of these FGD represent convenience samples of people in families affected by TB, to whom with had access because they or their family members were being served by the intervention. We recruited either during a treatment support visit to the household or by phone. We did not record the number of people who were approached who declined participation. However, study staff noted that the reason why the vast majority of participants were women was because most of the men they approached declined participation because of scheduling conflicts (i.e. they had to work during the day, when the FGD were scheduled to take place).

For the feedback FGD with health care workers, study staff approached health care workers in health facilities to invite them to participate. Although only doctors can prescribe preventive treatment, we invited both doctors and nurses because nurses are responsible for most of the day-to-day management of preventive treatment. While 8 health care workers representing 6 of the 9 health facilities that participated in the intervention agreed and gave consent to participate, only 5 health care workers representing 3 health facilities ultimately participated.

*FGD setting (COREQ item 14)*

FGD 1-7 were conducted in Spanish at the Socios En Salud office. FGD 8 was conducted virtually in Spanish via Microsoft Teams videoconference; a virtual FGD was necessary because of COVID-19-related restrictions on meetings.

*Non-participants (COREQ item 15)*

The lead author was present as an observer for FGD 3, 4, and 7. Co-author GSP was present for FGD 4 and 6. Some participants in FGD 3, 6, and 7 (caregivers) were accompanied by infant children. For FGD 8, in addition to the facilitators and participants, four Socios En Salud nurses working in the field team viewed the videoconference as observers.

*Description of sample (COREQ item 16)*

The description of the 8 focus group discussions (FGD), criteria for participation each FGD, numbers and sex of participants is described in the Methods section and Table 1 of the main text.

*Data collection (COREQ items 17-23)*

Guides for the three types of FGD are summarized below.

Planning FGD for members of families affected by TB on their preferences for treatment support during TB infection treatment, 30 minutes

| **Research questions** | **Probes** |
| --- | --- |
| Acceptability of community health worker visits or phone calls | Would you accept a community health worker visiting your home to offer counseling and support during TB infection treatment? Why or why not? If so, how often would you want this visit to occur? |
|  | Do you think that your neighbors or friends would accept a community health worker visiting their homes to offer counseling and support if they were taking TB infection treatment? Why or why not? |
|  | Would phone calls be preferable to visits? Why or why not? |
| Acceptability of family members as designated treatment companions | Would having a family member who is trained to offer you support during treatment help you complete your treatment? |
|  | If your neighbor or friend were taking TB infection treatment, how would they feel about having a family member offering them treatment support? |
| Acceptability of SMS reminders | How would you feel about receiving a daily SMS reminder that says “please remember to take your medications?” |
|  | How do you think your neighbors or friends would feel about receiving a daily SMS reminder? |
| Other ideas for adherence support | Do you have other ideas for how we can help people taking TB infection treatment to complete treatment? |

Feedback FGD for people who initiated TB infection treatment on their treatment experience, 60 minutes

| **Research questions** | **Probes** |
| --- | --- |
| Experience getting prescribed treatment and reasons for uptake | Tell me about your experience at the health center when the doctor told you that you should receive treatment for TB infection. |
|  | How well did the doctor or nurse explain to you the reason for treatment? Is there anything you feel that the doctor or nurse should have explained better? |
|  | What were your reasons for accepting treatment? |
| Barriers and facilitators to treatment adherence | What were the challenges you encountered during treatment? If not mentioned, ask about problems with side effects, picking up medications. |
|  | How did you remember to take the medications every day? (Or give the medications to your child, if participants are guardians) |
|  | Was there anything that you felt was helpful to you during treatment? For example, any people who were particularly supportive? |
| Ways to improve program | How can the services for treatment of TB infection be improved? |

Feedback FGD from health care workers who managed TB infection treatment on their experience, 60 minutes

| **Research questions** | **Probes** |
| --- | --- |
| Perceptions of TB infection treatment | How much awareness of TB infection treatment is there among your colleagues? |
|  | How important do your colleagues feel TB infection treatment is? |
|  | What are some of the doubts that your colleagues have about TB infection treatment? |
| Experiences managing TB infection treatment | What are some of the barriers you have encountered in managing TB infection treatment? |
|  | What are some things that have made it easier to prescribe treatment or manage treatment? |
|  | What are some factors that could cause a doctor to not prescribe TB infection treatment to someone whom the national guidelines say should receive treatment? |
| Ideas for improving TB infection treatment | What are some strategies that could improve the use of TB infection treatment among your colleagues? |
|  | Examples: formal trainings, one-on-one visits |

FGD were audio recorded and transcribed by a person who was not a member of the research team. Transcripts were not returned to participants for feedback. We did not repeat interviews with participants or take notes during FGD. Because the number of FGD and participants was predetermined by resource constraints, we did not use data saturation as a criterion for determining when to stop holding FGD.

*Coding (COREQ items 24-28)*

Coding was performed in Dedoose in Spanish by the lead author CMY and co-author GSP with guidance from JTG. The structure of the code tree was pre-determined based on the care cascade; themes were grouped by cascade step, and whether they were barriers and facilitators to completing that step. Individual themes were derived from the data. Participants were not asked for feedback on analytic results.

*Presentation of results (COREQ items 29-32)*

Major themes are presented in the Results section of the manuscript. We present not only the themes reflected by multiple participants’ statements but also individual experiences mentioned by specific participants that were not necessarily echoed by others. Supplemental table S4 presents supporting quotes for identified themes; a more concise version of this table is presented in the main text as Table 3.
